# “*My childhood affected my ability to be resilient in both good and bad ways*”: A mixed methods examination on the links between adverse childhood experiences, resilience, and transactional sex among young South African women

**DOI:** 10.1371/journal.pone.0341216

**Published:** 2026-01-28

**Authors:** Deborah Baron, Nisha Gottfredson O’Shea, Alexandra Lightfoot, Caroline Kuo, Sheri Lippman, F. Xavier Gómez-Olivé, Kathleen Kahn, Audrey Pettifor, Suzanne Maman

**Affiliations:** 1 Department of Health Behavior, Gillings School of Global Public Health University of North Carolina Chapel Hill, Chapel Hill, North Carolina, United States of America; 2 Sheps Center for Health Services Research, University of North Carolina Chapel Hill, Chapel Hill, North Carolina, United States of America; 3 RTI International, Research Triangle Park, North Carolina, United States of America; 4 Department of Health Studies, American University, Washington, District of Columbia, United States of America; 5 Division of Prevention Science, University of California, San Francisco, California, United States of America; 6 South African Medical Research Council/Wits University Rural Public Health & Health Transitions Research Unit (Agincourt), School of Public Health, Faculty of Health Sciences, University of the Witwatersrand, Agincourt, South Africa; 7 Department of Epidemiology, Gillings School of Global Public Health, University of North Carolina at Chapel Hill, Chapel Hill, North Carolina, United States of America; IFPRI: International Food Policy Research Institute, UNITED STATES OF AMERICA

## Abstract

**Background:**

South African women are disproportionately impacted by HIV. Among these women, adverse childhood experiences (ACEs) are common and have been linked to HIV risk behaviors, including transactional sex (TS). Resilience—or multi-level processes related to overcoming adversity—provides a strengths-based lens that may buffer effects between ACEs and TS.

**Methods:**

We conducted a convergent mixed methods study among women aged 18–25 years in Mpumalanga, South Africa. We used logistic regression to assess the association between ACEs and TS; and tested moderation effects of five resilience scales across social-ecological levels hypothesized to dampen the effect of ACEs on TS. In parallel, we conducted a photovoice study that utilized participant-generated images and narratives, and thematic and sequence analysis to explore how women exposed to ACEs perceive and use resilience to navigate TS relationships.

**Results:**

Our analysis included 1,222 women aged 18–25 years, of whom 714 (58.43%) reported ACE exposure, with 519 (42.47%) reporting 1–2 ACEs and 195 (15.96%) reporting ≥3 ACEs; 340 (27.82%) reported TS. Women reporting ACE exposure had increased odds of TS compared to those without ACE exposure, controlling for confounders (AOR = 1.52, 95% CI: 1.17–1.99, P = 0.002). Among women with histories of ACEs, women with ≥3 ACES had 2.55 times the odds of TS than those reporting 1–2 ACEs (95% CI: 1.79–3.63, P=<0.001). None of the resilience measures moderated effects between ACEs and TS. Through the photovoice study (n = 10), women described how ACEs necessitated resilience early in life; some applying it to avoid and others to sustain TS relationships.

**Conclusion:**

We examined the interplay between resilience, ACEs, and TS. Although quantitative results showed resilience did not buffer negative effects of ACEs on TS, the photovoice findings suggest resilience was salient and influential in women’s lives. Future research should explore resilience measures and interventions that address the complex gender and power dynamics that exacerbate women’s exposure to TS and HIV.

## Introduction

Each week, roughly 3,100 adolescent girls and young women (AGYW) aged 15–24 years living in sub-Saharan Africa acquire HIV [1]. In South Africa (SA), HIV prevalence is nearly double among females (20.3%) compared with males (11.5%) in what remains a hyper endemic country [2]. Among young women, HIV incidence is primarily heterosexually transmitted, spurred by inter-connected individual, social, economic, and structural factors that shape inequitable sexual relationships [3,4].

Transactional sex relationships are a complex, yet common, gendered practice that has been shown to contribute to young women’s high HIV burden in SA [5–7]. Distinct from sex work—transactional sex is defined as “noncommercial, non-marital sexual relationships motivated by the implicit assumption that sex will be exchanged for material support or other benefits” [8 p.193]. Transactional relationships occur within three inter-related paradigms characterized by distinct motivations: 1) meeting basic survival needs (food, shelter, and school fees) driven by poverty and limited earning opportunities; 2) seeking to elevate social status within society’s ever-increasing consumerism; and 3) desire for material exchanges as symbolic evidence of love and commitment by male economic providers, particularly within traditional patriarchal cultural norms [8]. Irrespective of motivation, emerging evidence indicates that women have varying degrees of agency around transactional relationships, particularly related to partner selection and when to start and end exchange relationships [9]. Critically, women’s agency appears to diminish during relationships, increasing women’s vulnerability to HIV as they lose power to negotiate condom use and maintain consensual, non-violent sexual interactions [10].

For many SA women, these precarious relationships might occur in the aftermath of having endured adverse childhood experiences (ACEs). Childhood trauma is ubiquitous worldwide and SA is no exception. A 2019 Soweto cohort study found that 88% of young women reported at least one ACE, and 34% reported four or more ACEs [11]. ACEs—characterized as traumatic events including physical, emotional and sexual abuse as well as exposure to dysfunctional family environments during individuals’ first 18 years—negatively affect the long-term wellbeing and health outcomes of adolescents and adults [12,13]. Two global reviews illustrate the links between ACEs, including co-occurrence and cumulative ACEs, and increased sexual risk behaviors, such as early sexual debut, condomless sex, transactional sex, and multiple sex partners [14,15]. Studies among young SA women corroborate these findings, showing ACE exposure was significantly associated with transactional sex [16], while another cohort study linked sexual abuse, emotional abuse, and physical punishment to higher HIV incidence [17]. Even controlling for the socio-economic realities of poverty (e.g., food insecurity, education completion) common to these settings, ACEs were shown to have a uniquely harmful and significant impact in shaping young women’s sexual health behaviors and outcomes.

Resilience—conceptualized as dynamic multi-level processes related to overcoming adversity—offers a strengths-based approach to examine protective factors that may decrease the links between ACEs and risky transactional sex [18,19]. Cultivated out of necessity, resilience processes are the ways in which people mobilize internal assets (e.g., adaptive and positive coping) and external resources (e.g., social support, community-level social cohesion) to negotiate and navigate their way through adverse environments [20,21]. Despite the increasing recognition of a socio-ecological approach to resilience, most resilience research continues to focus solely on individual level psychological frameworks [22]. There is an urgent need to conceptualize, measure and evaluate resilience promoting factors across multiple levels and assess how these co-occurring processes operate and potentially interact under stress to cultivate resilience [21]. In a systematic review of resilience-focused interventions aimed at preventing HIV and sexual risk behaviors among young women, LoVette et al. [23] found most of the studies operationalized resilience as individuals’ ability for adaptive coping, and thus, the majority of interventions targeted changes in individual sexual risk behaviors, such as condom use, harmful drinking, and multiple sex partners; none mentioned transactional sex. Despite inconsistent outcome measures, over half of these resilience-focused interventions reported improved sexual risk behavioral outcomes [23]. This review provides promising evidence about the use of resilience-based approaches in mitigating HIV risk behaviors. Yet, little is known about how ACE exposure may influence the associations between resilience and HIV risk behaviors, especially transactional sex. Further, while limited quantitative research is beginning to address links between resilience, social resources and transactional sex [24], there remain gaps in understanding these multi-level processes qualitatively and in the aftermath of young women’s childhood trauma. By investigating how ACEs may impact young women’s ability to use resilience strategies, this research draws much-needed attention to the unique prevention needs of women with histories of ACEs and contributes to the evidence base by expanding our understanding of the context in which resilience strategies are implemented.

### Study objective

We hypothesized that young women exposed to ACEs are more likely to engage in transactional sex, and that this positive relationship is weakened by resilience-promoting factors. To test the hypothesis, we conducted a mixed methods study using individual survey data, coincident community-level cross-sectional data, and qualitative data from a high HIV prevalence area of rural South Africa. The aims of this study were (1) to test the links between ACEs and engaging in transactional sex, and moderating effects of resilience factors on the link between ACEs and transactional sex, (2) qualitatively contextualize how young women exposed to ACEs understand and use resilience resources to mitigate and negotiate transactional sex relationships, and (3) integrate the data sets to gain a more holistic understanding of young women’s lived experiences of resilience within transactional sexual relationships and related risks to HIV acquisition.

## Methods

### Study design and setting

**Fig 1:**
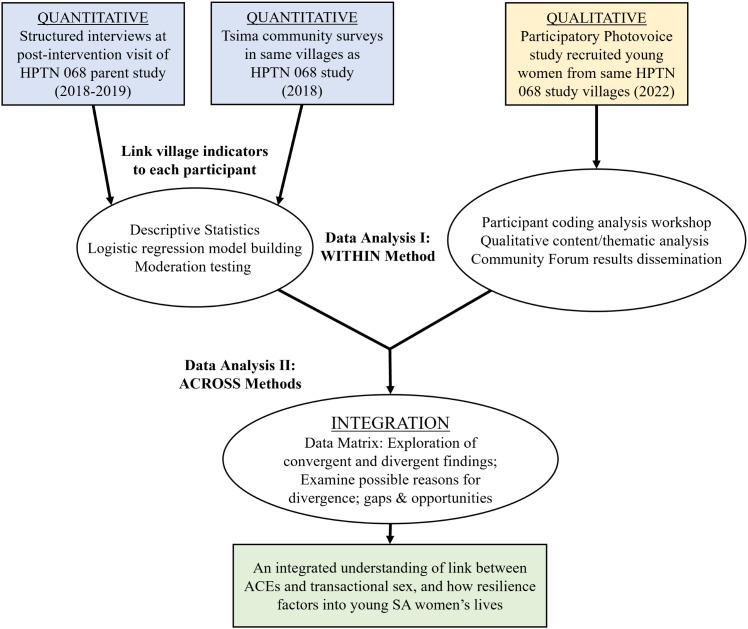
Data collection and analysis processes.

We used a convergent mixed methods study design [25], including a secondary quantitative analysis using survey data from two linked studies: HPTN 068 individual level post-intervention survey [26] and the Tsima Community Mobilization (CM) study’s community level survey [27], plus a separate qualitative photovoice study at the same study site with a distinct yet comparable sample. The quantitative and qualitative datasets were analyzed independently; after which we integrated results from each method to identify similarities and inconsistencies between the findings (Fig 1). To facilitate a deeper understanding of these complex phenomena and their interplay, the study design was grounded in the social ecological model (SEM) of health, and the theoretical frameworks of socio-ecological resilience, life course health development, and the bases of gendered power as adapted from social dominance theory [20,28–30].

The study was implemented within the SA Medical Research Council/ University of the Witwatersrand Rural Public Health and Health Transitions Research Unit that runs the Agincourt Health and Socio-Demographic Surveillance System (AHDSS) site in the rural Bushbuckridge sub-district in Mpumalanga province, SA [31]. Villages in this area were born out of the apartheid-led forced removal programs, resulting in lasting legacies of high levels of poverty, unemployment, and circular labor migration [31,32]. Within this socio-economic context, transactional sex relationships are common. Among young women in Bushbuckridge who participated in the HPTN 068 parent study, the mean age of sexual debut (vaginal or anal) was 14.7 years [6]. Further, 14% of sexually active young women (aged 13–20 years) reported previous transactional sex at baseline, with exposure to transactional sex linked to an almost three-fold increased odds of being HIV-positive [6]. Overall, HIV incidence in this cohort was 3.6% among 19-year-old women and rose to 4.3% among women 20 and older between 2011 and 2015 [26]. In 2022, Mpumalanga was ranked with the highest HIV prevalence of all provinces in SA—reported at 17.4% [33]. Within this high prevalence setting, AGYW face a disproportionate HIV burden, with 12% prevalence in Mpumalanga compared to 10.1% of AGYW nationally [34]. Moreover, ACEs are widespread in this region: a population-representative cohort study in Agincourt found that 55% reported at least one of the four ACEs measured, including 35% reporting physical abuse before age 16 [35]. Orphanhood, an ACE measured in this study, is also common in Mpumalanga, with 9.1% of the provincial population experiencing paternal loss and 3.1% maternal loss before age 18 [36].

Institutional Review Board approvals for the HPTN 068 Study (UNC IRB #16–0203), Tsima Community Mobilization Study (UNC IRB #14–2214), and all research activities for this mixed methods study, including the merging and analyzing of de-identified data sources and conduct of the photovoice study were obtained from the University of North Carolina at Chapel Hill (UNC IRB #21–0042). Ethics approval was also obtained from the University of Witwatersrand’s Human Research Ethics Committee (HREC IRB #320451, #101012), and Mpumalanga Department of Health. The Tsima Community Mobilization Study was additionally approved by the University of California, San Francisco (UCSF IRB #14–13575). The parent randomized control trial, the HPTN 068 Study (trial registration #NCT01233531), adhered to the Declaration of Helsinki’s guidelines for biomedical research involving human subjects. See S1 File for additional information regarding the ethical, cultural, and scientific considerations for this study specific to inclusivity in global research.

### Quantitative study

#### Data collection.

We conducted a cross-sectional secondary data analysis using logistic regression and moderation testing with data from two data sources collected during the final 2018–2019 waves of the respective studies. The first dataset included young women from the HPTN 068 study, a randomized controlled trial assessing cash transfers conditional on school attendance for HIV prevention among AGYW. The HPTN 068 study methods have been published elsewhere [26]. Briefly, females who met the inclusion criteria—including being aged 13–20 years, residing in the AHDSS villages, not pregnant or married, and enrolled in secondary school—were recruited between March 5, 2011 and December 12, 2012 [26]. Written consent or assent for young women and parent/guardian written consent (required if participant <18) was completed in the language of their choice (xi-Tsonga or English). Between 2011 and 2015, annual questionnaires were administered, available in the local language, xi-Tsonga, or English. Detailed information on socio-demographics, relationship history, sexual reproductive health and HIV prevention behaviors, and mental health was collected. Biological samples to determine HIV-serostatus were collected at each visit. Two post-intervention visits were conducted between 2015–2016 and 2018–2019, for which participants re-consented. For this analysis, inclusion was restricted to women aged 18–25 years who participated in the final 2018–2019 post-intervention visit and reported living in an AHDSS village at the time of this visit. A separate attrition analysis of the HPTN 068 cohort found no significant differences between participants who provided data for this last time point and those who did not in terms of baseline risk and protection factors or treatment arm [37].

Additionally, data was used from a 2018 cross-sectional population representative post-intervention survey of the Tsima CM Study, a cluster randomized-controlled trial that evaluated a community mobilization intervention designed to change gender norms and reduce HIV risk [27]. Between August –December 2018, 1,182 men and women AHDSS residents aged 18–49 years were sampled and written informed consent was obtained from all participants before administering the survey. Further details of the study design and results have been published elsewhere [38]. For this analysis, we used three scales from the CM population-based surveys that aligned with the theoretical conceptualization and framing of community-level resilience promoting factors and social resources.

#### Measures.

Transactional sex outcome: We constructed a binary ever/never variable, with participants classified as exposed if they reported engagement in any type of transactional sex. To create this lifetime measure, we aggregated data from each study and post-intervention visit where participants were asked yes/no questions about their three most recent sexual partners (e.g., *Did you feel like you had to have sex with [partner] because they gave you money? …because they gave you things?*). All marital relationships were considered non-transactional. Thus, participants were coded 1 if they reported ever engaging in any transactional sex with a non-marital partner at any study visit.

ACE exposure predictor: We constructed a dichotomous variable using validated measures from the WHO’s ACEs international questionnaire (ACE-IQ) that included 10 items spanning seven distinct ACE categories [39]. Participants were asked about five of the categories, including emotional, physical, and sexual childhood abuse and witnessing physical or emotional abuse in the household, that may have occurred during their first 18 years of life during the 2015–2017 post-intervention visit. Additionally, we created two separate measures for maternal and paternal death before age 18 by assessing responses during annual study visits about the death of a parent and the participant’s age at time of their parent’s death. If a participant’s parent died before age 18, they were classified as having experienced an adverse experience of maternal and/or paternal death during childhood (Full questions in S1 Table). Participants who self-reported ≥1 ACEs were reported as ACE exposed. To assess cumulative exposure and dose response, we calculated a summed ACE score (0–7) with a higher score indicating exposure to more ACE categories. Scores were categorized into three groups—0 ACEs, 1–2 ACEs and ≥3 ACEs—to align with previous research and enable comparison of findings [40,41].

Resilience moderators: Five scales were used to measure resilience-promoting factors at the individual (n = 1), interpersonal (n = 1), and community/village (n = 3) level. Each community-level variable represents a village’s average score across all survey respondents (regardless of demographics) using sample weights to match respondents to the target population of village residents [42]. All resilience measures were collected during the final 2018–2019 data collection waves.

*Internal Resilience*: We asked participants to self-rate their psychological resilience using the 25-item Connor-Davidson Resilience Scale (CD-RISC) [43]. The CD-RISC assessed individuals’ tenacity and self-efficacy, tolerance of negative affect, adaptability and secure relationships, and sense of purpose, control and meaning. Items score on a 5-point Likert scale with sum totals ranging from 0 to 100. Higher total scores reflect greater ability to cope with adversity and accept change positively [44]. The CD-RISC has demonstrated validity extensively, including among SA adolescents [45,46]. In this study, the Cronbach α was 0.94, indicating excellent internal consistency.

*Social Support*: We used the 10-item Social Provisions Scale to assess the extent to which participants reported familial and proximal resilience-promoting social environment resources were available to them [47,48]. Items covered: emotional attachment; advice or informational guidance; tangible help; opportunity for nurturance; and sense of belonging to a group [49]. Response options included a 3-point Likert scale. Summed scores ranging from 10 to 30 were calculated, with higher scores reflecting higher levels of social support. Cronbach α = 0.87 in this study.

*Social Cohesion*: This 6-item scale assessed people’s perceptions about their village’s community-level connectedness conceptualized as promoting individual resilience, including items about the extent members of their community trust each other, share values, and look out for one another [50,51]. Summary scores were calculated for each respondent, which were then aggregated to create a mean village score for each community.

*Community Consciousness*: This 17-item scale was used to assess how well village residents work together to address village-level issues and solve problems. Individual respondent summary scores were calculated, followed by an aggregated mean score for each village.

*Organizations and Networks*: This measure asked residents 11 yes/no questions about which types of community organizations (e.g., youth, church, cultural, etc.) support and work to improve people’s lives in their village.

Control variables: Based on prior findings from the parent study and preliminary analyses, we controlled for: age of participant; highest level of educational attainment grouped into three categories [(1) some secondary/high school education, (2) completion of Grade 12 matric/high school, (3) completion of tertiary education)]; and means-tested household social grant recipient as an indicator for socio-economic need [52]. Duplicative confounders (e.g., social grant receipt and food insecurity) were excluded to avoid multicollinearity and maintain parsimony.

#### Quantitative data analysis.

All statistical analyses were conducted in SAS v.9.4 [53]. Descriptive statistics was used to check for potential errors and outliers, to summarize the socio-demographic characteristics, and to estimate the prevalence and patterns of ACEs. We used chi-square tests to assess relationships between ACE group categories and demographic variables, and t-tests estimated associations between ACE exposure and mean resilience scores. Missingness was less than 1% on each of the variables used in the models; all models were estimated using complete cases. We used logistic regression to estimate unadjusted and adjusted odds ratios of the links between ACE exposure and transactional sex. A sub-group analysis model to estimate women who reported 1–2 ACEs vs. ≥ 3 ACEs was also fitted. All adjusted models controlled for age, education, and social grant receipt. To conduct the moderation analyses, we built our models via the following steps: 1) Linked each participant to their corresponding village-level measures based on their residence; 2) Mean-centered all continuous variables; 3) Fit separate adjusted multi-variable logistic regression models with each resilience measure added as a second predictor to ACE-exposure; and 4) Added the interaction term to assess moderation effects between ACE exposure and each individual resilience measure. Following Hayes’ [54] moderation testing approach, probing was planned for interactions that were statistically significant.

### Qualitative photovoice study

#### Study design and data collection.

In consultation with two local youth partners and a broader Stakeholder Working Group (SWG), we conducted a qualitative study using photovoice, a participatory group-based and visual methods approach, to examine how young women understand and experience resilience in the context of ACEs and how these formative experiences influence their sexual relationships and HIV prevention behaviors. Following a study design workshop and photovoice training with the SWG, we collaboratively developed a recruitment strategy that addressed the sensitive nature of recruiting women with ACEs histories. Women were recruited between June 23 – July 13, 2022. Youth partners and SWG members used flyers and word of mouth to conduct outreach through their professional and personal networks, referring interested individuals to field workers. To protect women’s privacy, field workers contacted potential participants via WhatsApp using discreet language (avoiding mention of research or HIV) to arrange an informational call. During these calls, field workers described the study and conducted screening using a standardized checklist. Participant inclusion included sexually active young women ages 18–25 years living in an AHDSS village who self-reported ≥1 ACE (same questions as quantitative analysis; S1 Table) and were going to remain in the area for the study duration. Those who met the inclusion criteria and expressed willingness to participate were invited to attend a group informational session and informed consent process. Women who chose to participate provided written informed consent in xi-Tsonga or English.

Five weekly study sessions took place between July 13-August 10, 2022. Sessions were facilitated in xi-Tsonga by seasoned field workers, and at times, the Principal Investigator (PI), an American and SA permanent resident, through the use of simultaneous translation. The PI and field workers each have extensive experience working in HIV with young SA women, which facilitated our ability to create a trusting environment conducive to sharing authentic stories and critical reflection. During orientation, we introduced the theme of resilience using culturally-relevant concepts like *Ubuntu*—an SA term meaning “I am because we are” and signifies communal resilience and interconnected strength—to describe the concept of community-level resilience. We also asked participants to define resilience themselves, with some using idioms, such as “She then decided to ‘*wear her big panties’* and started taking treatment” (P1) to describe the internal resilience of a friend after seroconverting [55]. All participants were loaned digital cameras and trained both in the mechanics of using the cameras and how to take photos safely, ethically, and with consent/assent, including obtaining signed media release forms before taking identifying images of any individuals (S2 and S3 File) [56]. To address potential social harm, a trusted social worker was identified by our youth partner for anyone wanting a referral.

Together with participants, we generated three photo assignments that aligned with the SEM approach used in the quantitative analysis. Weekly topics explored: 1) Impact of ACEs on psychological resilience and HIV prevention behaviors; 2) Interpersonal resilience (e.g., social support) and sexual relationships; and 3) How government facilitates young women’s resilience (through provision of social resources). Each week, participants took and shared a photo, followed by a group discussion using the SHOWED method – a structured series of sequential questions that utilizes participant-generated photos to facilitate critical reflection from a surface level understanding of the image to a societal root-cause analysis [57]. See S4 File for an example facilitation guide with tailored SHOWED questions. A final synthesis session in August and separate coding workshop with the participants in October 2022 helped guide the iterative analysis phase. Sessions were recorded with participants’ permission, and simultaneously translated and transcribed into English weekly to facilitate member checking at each subsequent session. Additionally, a locally trained third-party researcher fluent in xi-Tsonga and English checked the transcripts against the original audio recordings for accuracy and quality.

#### Qualitative data analysis.

We used thematic analysis and composite sequence analysis approaches to facilitate an understanding of how ACEs influence women’s life course development [58]. Steps in the analysis process included: 1) Preliminary read of all transcripts, conducted quality and member checks; 2) Developed codebook guided by photo assignments and thematic groupings from participants’ coding workshop; 3) Analyzed photos and transcripts, including the PI and a local researcher double-coding and developing summary and analytic memos in Dedoose, a web-based research software; 4) Discussed emerging thematic patterns and discrepancies, refined the codebook and finalized coding; 5) Developed code report summaries and matrices to further identify patterns and interpret findings. Beyond member checking, triangulation was achieved during a community forum and dissemination event in November 2023 that included participants, members of the site’s Community Advisory Board and the project’s SWG, Agincourt leadership, and local partners. With support from the PI and facilitators, participants presented the findings—sharing displayed photos accompanied by illustrative quotes. As such, we confirmed the results through a robust discussion between the study participants, community stakeholders and research staff attendees.

### Mixed methods data analysis

Following each independent analysis, we integrated the two datasets. We further applied an abridged version of Moffatt et al.’s mixed methods analytic framework to examine inter-method discrepancies by investigating the rigor, outcomes, and comparability of the individual study components [59]. Through this process, we expanded our findings to provide holistic insights greater than the sum of each methodological part.

## Results

### Participant characteristics

A total of 1,222 women aged 18–25 years (*m* = 22.79 years) were included in the quantitative analysis, of whom 714 (58.43%) reported at least one ACE exposure, 519 (42.47%) women reported 1–2 ACEs and 15.96% of the total sample (*n* = 195) reported ≥3 ACEs; 340 (27.82%) reported ever in engaging in transactional sex. Table 1 shows the distribution of socio-demographic variables and prevalence of transactional sex for the sample and each ACE category. Reporting ≥3 ACEs was significantly linked to education level achieved, where 27.18% of women with ≥3 ACEs did not complete secondary school compared with 19.88% of women with no ACEs and 15.61% with 1–2 ACEs (χ² (2, N = 714) =13.82, p = 0.001). A larger percentage of women with ≥3 ACEs were living with HIV and lived in households supported by social grants than those with ≤2 ACEs, but these associations were not statistically significant. The mean CD-RISC resilience score was 68.15 (*SD*: 14.57) with no difference in mean resilience scores between women who reported any versus no ACE exposure. However, among the sub-set of women with any ACE exposure, mean resilience scores were significantly lower (3.29 points, p = 0.01) for women with ≥3 ACEs (*n* = 195, *m* = 65.58 points) than with 1–2 ACEs (*n* = 519, *m* = 68.87 points), suggesting a dose response between cumulative ACEs and resilience. Social support mean scores were comparable across sub-groups.

**Table 1:** Participant characteristics of young SA women in rural Mpumalanga, Aged 18-25 years, 2017-2018 (N=1,222).

		Experienced ACEs (n=1,222)		Experienced ≤1 ACEs (n=714)	
	Overall Sample *(N*=1,222) Mean (*SD*) Frequency (%)	No ACEs *(n=508)* Mean (SD) Frequency (%)	≤1 ACEs *(n*=714) Mean (*SD*) Frequency (%)	1-2 ACEs *(n*= 519) Mean (SD) Frequency (%)	3 or more ACEs *(n*=195) Mean (SD) Frequency (%)
**Adverse Childhood Experiences (ACEs)**		508 (41.57%)	714 (58.43%)	519 (42.47%)	195 (15.96%)
**Age, years**	22.79 (1.46)	22.83 (1.45)	22.76 (1.47)	22.75 (1.42)	22.79 (1.60)
**Marital status**					
Never married	1,084 (88.70%)	452 (88.98%)	631 (88.38%)	455 (87.67%)	176 (90.26%)
Married	127 (10.39%)	50 (9.84%)	77 (10.80%)	58 (11.18%)	19 (9.74%)
Divorced/Separated	11 (0.90%)	5 (98.42%)	6 (0.84%)	6 (1.16%)	0 (0%)
**Highest level of education**					
Some secondary education/high school	235 (19.23%)	101 (19.88%)	137 (18.77%)	81 (15.61%)	53 (27.18%)
Completed matric/high school	716 (58.59%)	289 (56.89%)	427 (59.80%)	328 (63.20%)	99 (50.77%)
Completed tertiary education^a^	271 (22.18%)	118 (23.23%)	153 (21.43%)	110 (21.19%)	43 (22.05%)
**Household social grant receipt**					
Yes^b^	1096 (89.69%)	450 (88.58%)	646 (90.48%)	466 (89.79%)	180 (92.31%)
No	126 (10.30%)	58 (11.42%)	68 (9.52%)	53 (10.71%)	15 (7.69%)
**Serologically verified HIV status**					
Living with HIV	113 (9.25%)	45 (8.86%)	68 (9.52%)	45 (8.67%)	23 (11.79%)
Not living with HIV	1109 (90.75%)	463 (91.14%)	646 (90.48%)	474 (91.33%)	172 (88.21%)
**Resilience processes**					
Individual: CD-RISC (score range: 0-100)	68.15 (14.57)	68.40 (14.66)	67.97 (14.50)	68.87 (14.35)	65.57 (14.68)
Social support (score range: 10-30)	27.87 (3.19)	27.89 (3.21)	27.86 (3.17)	27.84 (3.20)	27.92 (3.12)
**Have had transactional sex relationship**					
Yes	340 (27.82%)	118 (23.23%)	222 (31.09%)	130 (25.05%)	92 (47.18%)
No	882 (72.18%)	390 (76.77%)	492 (68.91%)	389 (74.95%)	103 (52.82%)

^a^Tertiary=Grade 12/Std 10 and post-secondary school level diploma, certificate, or college degree (e.g., bachelors, masters, PhD).

^b^Social grant break-down (n=1,096) reported: Childcare support (n=993, 90%); old age pension (n=316, 29%); foster care support (n=27, 2.46%); disability (n=28, 2.5%). Participants could select more than one.

For the qualitative study, we recruited ten women aged 19–25 years to participate in the photovoice study. The average age was 23 years, with nine participants reporting never being married and one woman in a customary marriage, which are lawful unions governed by indigenous African customary law in SA [60]. These demographics are comparable to the quantitative sample. ACEs history for both cohorts are reported in Table 2.

**Table 2:** ACEs self-reported by participants in quantitative and qualitative studies.

	Quantitative	Qualitative
Adverse Childhood Experiences (ACEs)^a^	*N*=714 Frequency (%)	*N*=10 Frequency (%)
No. of ACEs (average among women with ACEs)	2.04	3.6
Dad died before 18	350 (49.021%)	2 (20%)
Mom died before 18	161 (22.55%)	4 (40%)
Experience verbal/emotional abuse	294 (41.18%)	9 (90%)
Witness verbal/emotional abuse	286 (40.06%)	7 (70%)
Experience physical abuse	158 (22.13%)	4 (40%)
Witness physical abuse	159 (22.27%)	5 (50%)
Child abuse, including severe beatings and sexual abuse^b^	48 (6.72%)	5 (50%)

^a^Items began with prompt, “When you were growing up, during the first 18 years of your life….” with exception of parental death, which asked for specific age at time of ACE.

^b^This was the only survey variable with substantive missingness, possibly due to the sensitivity of including sexual abuse—defined as “sexual acts with someone 5 years older than you while you were younger than 18” within the query.

### Quantitative findings

Table 3 reports results from the unadjusted and adjusted analyses of the links between ACEs and women’s engagement in transactional sex. In the unadjusted model, results indicate that young women with ACE exposure had increased odds of ever engaging in transactional sex than those with no ACE exposure, (OR=1.49, 95% CI: 1.15–1.93, P = 0.003). When controlling for age, education, and receipt of social grants, women who reported at least one ACE had higher increased odds of ever engaging in transactional sex than those with no ACE exposure, (AOR = 1.52, 95% CI: 1.17–1.99, P = 0.002). Among the 714 women with ACE exposure, women with 3 ≥ ACES had over two and a half times the odds of transactional sex than women who reported 1–2 ACEs, controlling for age, education, and social grant, (AOR = 2.55, 95% CI: 1.79–3.63, P=<0.001). Table 3 also shows results from the adjusted moderation analysis on the association between ACE exposure and transactional sex, and the interaction effect between ACE exposure and each of the five resilience-promoting variables hypothesized to buffer the link between ACEs and transactional sex. In each of the fitted models, results show that none of the resilience measures moderated the effects between ACE exposure and transactional sex in this sample.

**Table 3:** Estimated main effects and moderating effects of resilience-related moderators associated with transactional sex and ACEs.

	Exposure / Interaction	Transactional sex^a^			
Model		Estimate	OR/AOR	95% CI	p-value
0	ACE-exposure vs. no exposure (unadjusted model)	0.40	1.49	1.15-1.93	0.003*
1	ACE-exposure vs. no exposure	0.42	1.52	1.17-1.99	0.002*
2	3≥ ACEs vs. 1-2 ACEs	0.93	2.55	1.79-3.63	<0.001*
3	ACE-exposure	0.42	1.52	1.17-1.99	0.002*
	Psychological resilience^b^	-0.13	0.88	0.71-1.07	0.21
	ACE-exposure * Psy resilience	0.04	–	–	0.74
4	ACE-exposure	0.42	1.52	1.17-1.99	0.002*
	Social support^b^	-0.005	0.99	0.81-1.22	0.96
	ACE-exposure * social support	0.14	–	–	0.29
5	ACE-exposure	0.42	1.53	1.17-1.99	0.002*
	Community consciousness^b^	0.62	1.86	0.54-6.44	0.33
	ACE-exposure * community consciousness	0.2	–	–	0.81
6	ACE-exposure	0.43	1.5	1.18-2.01	0.002*
	Social cohesion^b^	0.57	1.76	0.50-6.26	0.38
	ACE-exposure * social cohesion	0.22	–	–	0.78
7	ACE-exposure	0.42	1.52	1.65-1.98	0.002*
	Orgs and networks^b^	-0.25	0.78	0.27-2.26	0.65
	ACE-exposure * orgs and networks	0.74	–	–	0.28

^a^Models 1-7 controlled for age, education level completed, and social grant recipient.

^b^Main effect reported for variable when ACE-exposure=0

* p-value ≤0.01 (statistically significant).

### Photovoice findings

Our analysis of the photo discussions uncovered how childhood traumas put SA young women on a path to increased risk for transactional sex relationships. While the moderation testing did not show significant buffering effects, the photovoice findings revealed striking nuances in how the participants’ varying perceptions of resilience in relation to transactional sex served to protect, and at times, exacerbate young women’s exposure to these precarious relationships.

During our orientation session, the group discussed their understanding of resilience and opted to use the xi-Tsonga word, *Kuiyisela,* to convey resilience, which directly translates to “*hold on*” or “*to persevere.*” (P10) Participants viewed resilience primarily as an internal process:

“*To me resilience at a personal level means being able to cope with difficult situations, like not giving up even when life becomes hard. To me resilience means to remain strong even in difficult circumstances.*” (P1)

Yet, when examining resilience within the context of transactional relationships, participants often described divergent approaches and consequences in terms of how women perceive and utilize resilience via varying coping mechanisms, relationship tactics, and adaptive strategies to adversity. We explore these complex associations and tensions in the sections that follow.

#### ACEs and their effects instilled resilience at an early age.

**Fig 2:**
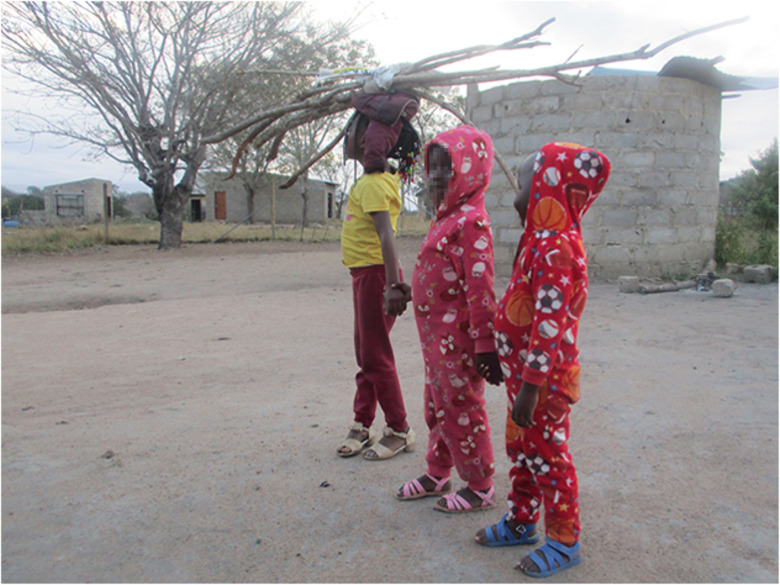
“*My struggle*”.

Participants described the myriad material, psychological, and health effects of ACEs on their life trajectories. One salient theme highlighted the ripple effects of maternal death, which often left participants food insecure, lacking stable housing, and vulnerable to physical and emotional abuse and neglect by extended family and alleged caregivers. These struggles led girls to take on the primary caregiver role for their younger siblings, as they sought to protect them from the same harmful exposures. The photo in Fig 2 was taken to capture the weight of the daily realities due to neglect and orphanhood:

*“Life was hard for me because the situation I was in forced me to mature faster than my actual age. I had to take care of my siblings at the age of eight, I was a child myself but a mother to my two siblings. My father also did not care about us, it’s like he almost forgot that we existed…I’m showing that young child who is carrying wood on her head to demonstrate my experience, the wood represents the burden that I was carrying. I was forced to carry huge responsibilities that were beyond my abilities at a young age.”* (P6)

For the women who lacked parental support and protection, resilience was frequently associated with self-reliance:

*Most of the time I would be alone taking care of myself while my mother was away [working in another city]…I was forced to learn to cook for myself at a young age…I had challenges both at home and at school, I had to face these tough challenges at an early age but because I knew what I wanted, I became resilient. I learned to take care of myself and I did not give up on going to school regardless of the challenges I was facing.”* (P1)

Beyond stories of overcoming obstacles to meet their basic survival needs, participants also spoke of the psychological toll of ACEs. Women described the emotional fallout, including anger, depression, and suicidal thoughts, caused by their trauma. For P6, who faced a series of spiraling abuses following the death of her mother, she described it as:

“*this anger is like an animal that lives inside you, an animal that needs to be constantly fed and when it’s hungry, you go crazy.*” (P6)

Others described how ACEs compelled them to become withdrawn and resist forming new attachments:

“*I grew up a bubbly child, I loved meeting new people and socializing but now I’m no longer open and welcoming to people…I probably did not deal with my trauma because I no longer have friends and I have become more of a loner.*” (P1)

Of note, some participants expressed how emotional abuse and witnessing abuse inflicted longer lasting pain and deeper wounds to heal than physical abuse:

*“Watching your mom getting abused by the man that claims to love her can affect you in a negative way. From my experience, emotional pain takes [more] time to heal compared to physical pain.”* (P2)

**Fig 3:**
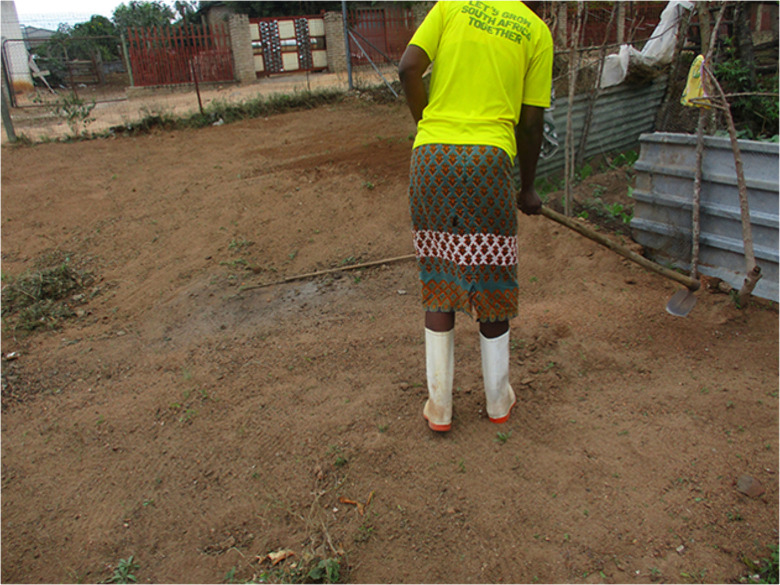
“*Let’s Grow South Africa Together*”.

In contrast to stories of household abuse and neglect, a few participants credited their parents’ positive parenting for teaching them how to cope and adapt in the face of life adversities. Fig 3 represents one participant’s appreciation for her own mother’s perseverance by depicting her mother gardening in what seems to be unfertile dirt. Despite a life of hardship, her mother remains determined to cultivate the land, symbolizing her hope for her daughter’s future. The photographer noted: *“this picture demonstrates that my mom sacrificed a lot for me to have the life that I have today.”* (P10) Only one woman spoke of being raised in a two-parent household and its positive impact:

*“Growing up was difficult but I’m grateful for my parents because they always taught us how to deal with life challenges…they were very open to talk to us about everything…My parents instilled confidence and independence in me. As a result, I can face any difficult situation that I come across with every day…I am resilient because my past experiences made me the stronger person that I am today.”* (P8)

As participants reflected on the impact of ACEs on their lives, they reckoned with the complexity of the consequences: *“my childhood affected my ability to be resilient in both good and bad ways.”* (P1) While their resultant self-reliance and perseverance were highly admired life skills they benefited from, they often came at the cost of their mental well-being, which in turn, had lasting effects on their sexual relationships as they aged into young women.

#### Resilience helped women avoid and also sustain transactional sex relationships.

**Fig 4:**
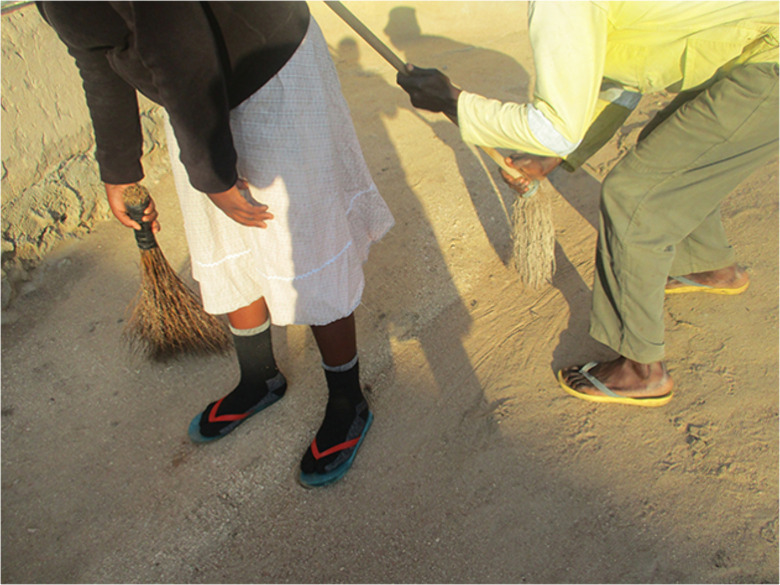
Man beating woman while she cleans.

Hardship and deprivation were the backdrop for many participants’ narratives about ACEs, resilience, and transactional sex relationships. Longing to escape poverty, some participants described transactional sex as a worthwhile path to what women described as a “*soft life*” (P1, P2, P5) – a lifestyle beyond mere economic survival or security, but also inclusive of material and economic ease. Yet, this lifestyle often had consequences that included abuse, with one participant declaring that being with “*a sugar daddy means accepting to be abused.”* (P6) Several participants’ photos captured this idea of enduring intimate partner violence (IPV) within transactional sex relationships, such as Fig 4 which poignantly depicts a woman being beaten by her partner as she does household chores. The participant shared:

*“I took this picture because it shows some resilience… Even though she is being abused she is not thinking of leaving the house because she thinks that [he] loves her. She doesn’t even know where to go so she rather stays.”* (P4)

This example highlights how some women perceived resilience as perseverance to tolerate abuse for the sake of economic survival and optimally a “*soft life*” of material comfort. Participants used this term commonly; many believing that women in their community would *“rather suffer in a mansion with all the nice things [they] want than having peace at the shack”* (P6) and *“as long as you wake up to warm bacon [all laugh].”* (P10)

In contrast, other participants were adamant that resilience entailed taking actions that prioritize bodily (physical and mental) self-preservation and health over material benefits or social status. One participant summed up this view during member checking, noting that accepting abuse reflects a lack of resilience:

*What I was saying is that* ***I’m not going to endure the pain of being abused, I think there is no resilience*** *[among women who accept abuse] but, on her [P4 as cited above] statement, resilience is there, as she rather stays than going back home to poverty. She accepts being beaten up at the end of the day because she wants to have access to a good lifestyle… like having access to a cold drink. [However,] I would rather leave than to be beaten up and abused but she accepts to be abused for the sake of living ‘soft life’.* (P2, emphasis added)

For P2 and others, they disagreed with the notion that enduring abuse for economic benefit signifies resilience. Instead, they attributed their resilience cultivated during childhood as providing the inner strength and determination to navigate out of unhealthy relationships and break their cycle of abuse, even if it meant sacrificing economic security or desirable lifestyles:

*“I don’t want to endure pain for the sake of a relationship. I will end a relationship that threatens my physical or emotional health any day, any time. I have been resilient my whole life.”* (P6)

The desire to minimize further trauma was additionally characterized through examples of emotional detachment with sexual partners. Women were keenly aware these relationships served a transactional purpose and accepted them as such. The same participant continued, describing how she cycles through boyfriends, using relationships for gifts and to be able to afford socializing. Even if the men become emotionally attached, she does not:

*“I cannot show affection towards my sexual partner. I feel like I am emotionally unavailable for the relationship. I am not mentally invested, and it feels like I’m in that relationship for fun and nothing serious… He honestly tries to show me love but I can’t love him back. It was similar situations with my ex-boyfriends... I lost my mother, so it doesn’t hurt when men leave me.”* (P6)

**Fig 5:**
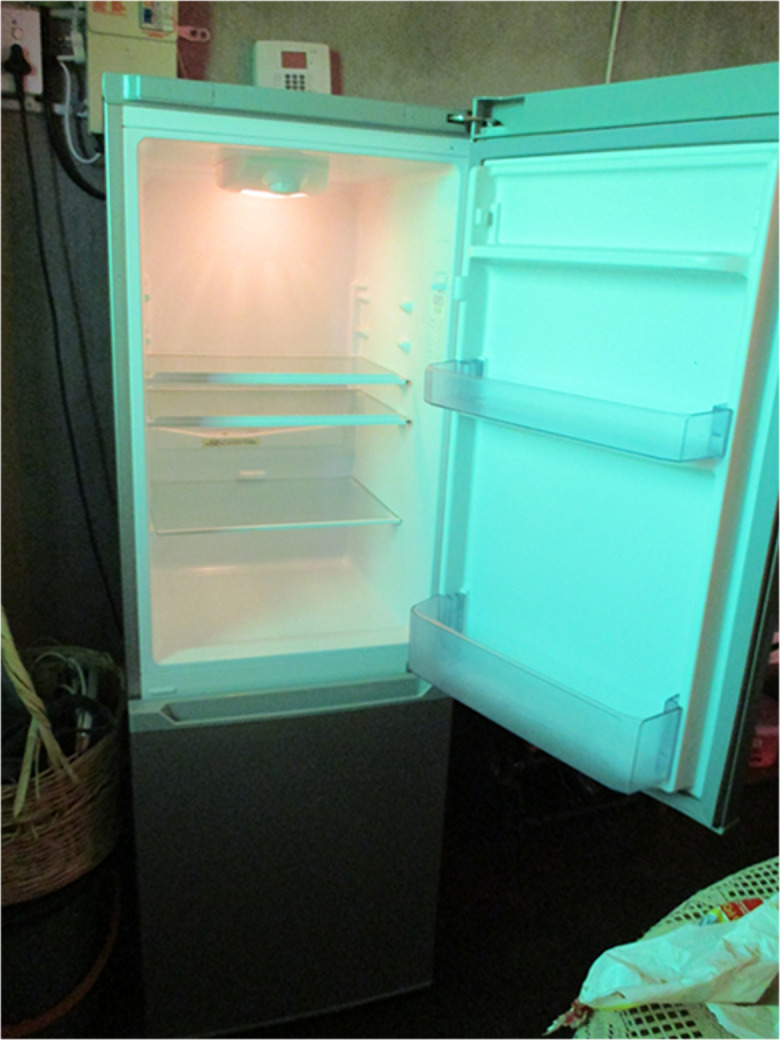
“Month End”.

Even among the non-violent relationships, women discussed links between childhood trauma and subsequent mistrust: *“I think [trauma] affects us negatively because of the lack of trust…I am also in a healthy relationship, but I do not trust my boyfriend”* (P10). Another participant described her willingness to remain in a loveless transactional relationship as enacting resilience, given the financial support it affords her children. She depicted her poor living conditions in Fig 5, an un-staged image of her empty fridge that she entitled “*month end*” highlighting the eager wait for her partner’s first of the month paycheck to buy groceries for her children:

P9:“*I also stayed in a relationship with the father of my baby because I needed financial support to take care of the baby. The relationship was not working but I couldn’t break up with him.”*

F2:“*How does that relate to resilience?”*

P9:“*I was resilient because I didn’t love him, but I had to stay with him because I needed his money…I had no other choice.”*

Conversely, some women adamantly avoided transactional relationships despite the temptations of the economic relief it would afford. For them, resilience manifested through their steadfast resistance to transactional sex and the feared consequences it may incur. For instance, one woman who had suffered multiple ACEs (n = 5) and had to leave school at an early age to care for her siblings while her father worked in another province, relished that:

*“What makes me happy is just because I never involved on the situations [i.e.,transactional sex] that make myself to live a soft life while it will end in tears or the things that I should regret at the end.”* (P5)

Another participant recounted living next door to her neglectful father who re-married:

*“Sometimes I would see my father eating nice food while I did not have food to eat. The sad thing is that my father was not taking care of me…This was a painful experience for me, but I had to be resilient because I did not want to end up doing bad things like sleeping with men so that I can live good life like my father’s children. I can say that I am where I am today because of resilience.”* (P4)

As a whole, these examples illustrate the complex, and at times, conflicting perspectives women had on resilience and its role in facilitating outcomes; some taking resilient action to minimize exposure to transactional sex relationships and subsequent HIV risk and related health consequences while others entered and sustained transactional relationships for the more immediate economic benefits.

#### Lacking social resources following ACEs left women vulnerable to transactional sex.

Another strong theme was the absence of parental and social support growing up—often a direct result of being orphaned, neglected, abused—which combined with insufficient sexual education in schools left women vulnerable to unsafe transactional sex. Several participants commented that *“we lacked people who could teach us about the consequences of having sex, like getting HIV or falling pregnant at an early age.”* (P2) One participant further described how parents use physical abuse in lieu of informational support and guidance that could help young women better navigate sexual relationships as they transition to young adulthood:

*“Our parents failed to tell us the truth. Instead of them telling us the truth, for instance let’s say I slept at my boyfriend’s place and come back home in the morning, my mom would opt to beat me up instead of teaching me about the consequences of my wrong doings. I think that is what has affected us during childhood…Most of us ended up becoming mothers at an early age because of that.”* (P3)

Peers often filled these informational voids and encouraged transactional sex by focusing selectively on the benefits and omitting the risks: *“our peers will only tell you that it is fine to do it [transactional sex]. They will talk about the exciting part only, but they will not tell you about the consequences of it.”* (P6) Blurring the lines between peer pressure and peer support, participants described the practice of “*tagging along*” where women play match maker to help their friends secure transactional sex partners:

*“Let’s say I am in a relationship with a rich guy who does everything for me. My friend on the other side has a boyfriend who does not afford material things…Because we are friends when they ask things from me, I will tell them to ‘tag along’ whenever I go out with my boyfriend so that they can meet his friends who are also rich. In this way, my friends will be pressured to have relationships with my boyfriend’s friend because of the pressure of wanting to be like me. Most girls are in transactional relationships because of pressure from their friends.”* (P1)

Importantly, a few participants acknowledged the resilience-promoting role of poverty alleviation and social protection programs for meeting their economic needs. Citing social grants and home-based care specifically, these participants credited these programs with reducing women’s reliance on transactional sex relationships with “*blessers*”, a colloquial SA term used to refer to older, often married wealthy men who “bless” younger women with gifts, money, and other gains in exchange for sexual favors:

*Many households lack basic needs such as food, but the government has tried to address some of these challenges through home-based care. [They] support people with food and some also provide school uniforms for those in need, especially young women so that they do not go around looking for support from blessers.“* (P9)

Yet, despite these social provision measures, most participants felt they were on their own when it came to navigating life challenges, having become accustomed to not having social support during childhood. When asked about how non-governmental organization and village-level community resources help women prevent HIV, one participant replied simply, *“I don’t relate with the community [so] it’s difficult to think about this question.”* (P2) Thus, while community resources were often perceived as non-existent or inaccessible, transactional sex relationships were readily within reach and available to women.

## Discussion

This mixed methods research is among the first to investigate how the links between childhood trauma and transactional sex are influenced by resilience among young women in rural SA. Quantitative and qualitative findings showed convergent results indicating important links between ACE exposure and having ever engaged in transactional sex. Yet, our examination of resilience revealed divergent results, with moderation testing showing that none of the resilience measures buffered the negative effects between ACEs and transactional sex. Meanwhile, findings from our photovoice study show resilience to be a complex and salient phenomenon, in which its role as a moderator between ACEs and transactional sex is non-linear, and at times, contradictory.

Transactional sex was common in our study, with 27.82% of participants reporting having ever engaged in transactional relationships. This finding corresponds with research across diverse rural and urban sub-Saharan African settings showing transactional sex is prevalent among young women. HIV prevention studies with similar populations show a range of comparable prevalence: 22% among women ages 15–25 in a cohort in Malawi [61], and 42.6% past-year prevalence among SA women ages 18–30 in informal urban settlements [16]. More broadly, national adolescent surveys from four African countries found that 36% (Burkina Faso), 75% (Ghana and Uganda) and 80% (Malawi) of sexually active adolescent girls ages 12–19 reported ever having transactional sex; in contrast to the substantially fewer adolescent males (5% − 35%) who reported transactional sex [62]. These patterns reenforce the need for addressing the harmful gender norms, structural inequities, and limited economic opportunities that relentlessly compel young women toward transactional relationships.

Exposure to ACEs was also widely reported in our survey data, with nearly sixty percent (58.43%) of the sample experiencing at least one ACE and 15.96% experiencing 3 ≥ ACEs. This finding is consistent with the seminal ACE Study and global burden of violence against children [12,13]. Regional studies have found even higher prevalence of ACEs; a recent pooled analysis from five PEPFAR-participating sub-Saharan countries reported 75% of females having experienced at least one ACE and 21.3% experienced three or more [40]. Similar to a recent Soweto cohort study, which found 88% of women reported at least one ACE [11], these studies’ higher prevalence findings can be attributed to their inclusion of additional household adverse events, including substance abuse, mental illness, incarceration, and unemployment, as well as counting witnessing community violence toward the overall ACE score. Although our survey analysis did not measure these broader adverse experiences, our qualitative findings suggest that community violence and chronic unemployment are pervasive in this rural setting. These convergent findings underscore that childhood abuse remains a pressing issue in SA, and further raises important questions for developing multi-level interventions to prevent childhood abuse and adversities across individual, household, and community levels.

Adding to the growing evidence indicating links between ACEs and transactional sex, our findings showed a significant association between ACE exposure and odds of engaging in transactional sex, and this association was more profound among women with 3 ≥ ACEs. In Kanagasabai et al.’s pooled analysis, they found higher odds of women’s transactional sex in the past 12 months were linked with specific ACEs, including childhood sexual violence, witnessing IPV, and witnessing violence in the community [40]. Another cross-sectional study with women living in urban informal settlements in SA similarly found significant links between emotional, physical and sexual forms of childhood abuse and transactional sex [16]. Our study contributes to better understanding these findings, as it is the first to our knowledge to also examine these relationships qualitatively. Through participant-generated photos and narratives, key themes emerged revealing how orphanhood and neglect result in girls’ loss of parental social, emotional, and informational support, especially with regards to sex education—known protective factors in helping young women refrain from sexual risk behaviors, including transactional sex [63]. Meanwhile, emotional and physical abuse had lasting psychological effects, including depression, which recent research has quantitatively linked to developmental trajectories of increased transactional sex [64]. Our qualitative findings help illustrate the nuanced ways in which these complex and non-linear developmental processes affect girls’ life course trajectories and worsen their already high vulnerability to the widespread practice of transactional sex.

Examining resilience in the context of the multi-level adversities faced by people at risk of HIV offers a promising lens to determine the resources that may promote positive health behaviors (including sexual health) and buffer against life traumas [22,23]. Despite the growing recognition that resilience entails multi-component processes, most research continues to measure resilience solely at the individual level with limited inclusion of relational resilience. Our study sought to address this gap with a mixed methods analysis of resilience-promoting factors across three social ecological levels that may weaken the links between ACEs and transactional sex. In the survey analysis, none of the five resilience factors (psychological resilience, social support, networks, social cohesion, community consciousness) we tested moderated the links between ACEs and transactional sex. However, the qualitative findings uncovered women’s varied perspectives and showcased the contrasting and consequential ways women enact resilience to both avert and also endure transactional relationships depending on their life circumstances and priorities. If resilience was associated with transactional sex in two opposing directions, this may have washed out any moderation effects and explain our divergent findings. To understand and expand upon these findings, we compared the convergent and divergent inter-method results and discovered four essential insights.

First, convergent findings suggest childhood trauma had immediate and lasting impacts on young women’s resilience. In our survey, individual resilience scores were consistent with other SA studies in youth populations [45,65,66]. Our findings additionally showed that among women exposed to childhood trauma, those with lifetime exposure of 3 ≥ ACEs had significantly lower mean resilience scores than women with 1–2 ACEs. These findings build on two recent large cross-sectional studies among adults in high-income countries that similarly show a dose response relationship between cumulative ACEs and resilience also using the CD-RISC [67,68]. While these studies included adults 18–69 years across the life span, our study highlights this association can occur early in adulthood—which may have life-long implications for women’s health trajectories. Even as some critique the CD-RISC for being limited to assessing individual personality and coping traits, this concept of resilience aligns with the photovoice participants’ descriptions as an individual and internal phenomenon reflecting one’s hardiness, coping skills, and ability to deal with acute and chronic stressful situations.

Second, the integration of divergent results allowed us to combine the perspectives of women participants (insiders) and researchers (outsiders) to better understand how resilience is associated with transactional sex in both positive and negative ways depending on how one thinks about the practice. In the book, Quantity and Quality in Social Research, Bryman observes a key “distinction between quantitative and qualitative research is that the former is orientated to the specific concerns of the investigator and the latter to subjects’ [participants] perspectives” [69 pp140–141]. Thus, we as HIV researchers primarily associate transactional sex with the negative outcome of HIV risk (and at times, acquisition) while many of the women participating in the qualitative study associate transactional sex and resilience with another outcome—economic comfort and the relief from chronic deprivation. Literature has shown that young women are able to exert agency within transactional relationships [9,70], exhibiting “considerable decision-making control over the process of relationship formation and termination” [9 p12]. These studies contribute valuable insights to how women enact upon their motivations to engage in transactional sex, yet give little attention to how ACE exposure may influence female agency in these often-precarious relationships. For example, our photovoice findings on women’s reported psychological effects of ACEs—including mistrust, emotional detachment, and communication challenges—align with a growing area of research on developmental trauma disorder, a condition that extends beyond post-traumatic stress disorder (PTSD) and has been significantly linked with children’s traumatic separation from a primary caregiver and emotional abuse, when controlling for other PTSD symptoms [71]. Our findings showed how these effects impaired participants’ ability for healthy intimate sexual relationships, and suggest women used transactional sex relationships as an alternative that met dual purposes. Firstly, transactional sex helped them achieve a ‘*soft life*’ – an aspirational lifestyle free of the stressful financial uncertainty and material struggles that characterized their childhoods. Additionally, transactional relationships did not require a secure emotional attachment that many felt incapable of. These findings build on previous research that has shown, “[c]hildhood emotional abuse is associated with insecure attachment and identity, intimacy, empathy, and self-direction problems in adolescence” [71 p717]. More research is needed to better understand these pathways, and the links between childhood traumatic events and transactional sex, especially where IPV is involved. A separate path identified through photovoice highlighted women’s resourcefulness, particularly through accessing social services, as an active resilience process to minimize enticement to transactional sex. Roughly 90% of our quantitative cohort reported receiving a means-tested social grant, indicative of this population’s low socio-economic status. However, these grants are insufficient to cover basic expenses, often forcing caregivers to choose between meeting nutritional or education needs for their children [52]. Within the public health and resilience fields, there are unique opportunities to investigate expanded social protection programs, and contribute to the growing body of evidence showing these programs can reduce poverty, dependence on men for economic security, and sexual risk practices, including unprotected sex and transactional sex [72,73].

Third, women’s varying conceptualization of resilience in this study raises important questions for how to measure resilience in HIV research. In a review of resilience and HIV research, Dulin et al. [22] found that the majority of HIV research uses general resilience measures (e.g., unlike HIV stigma measures developed distinctly with HIV determinants in mind), and most of these measures only address a single level of the SEM of health. We addressed one gap by using a combination of single-level measures to assess resilience resources at different social ecological levels; however, none of these measures were gender-sensitive nor HIV-specific. Developing outcome-specific resilience measures that identify and evaluate protective and risk determinants specific to young women’s lived realities that put them at risk for HIV could inform the development of targeted HIV prevention resilience-strengthening interventions. As discussed above, social resources may also play a role in promoting women’s resilience, and thus, measuring instrumental support in addition to other types of social support could prove valuable for better understanding this phenomenon and informing intervention development. Such programs would serve to leverage women’s protective skills to negotiate assets and navigate resilience resources to facilitate safer sex practices within transactional sex relationships, rather than endeavoring to eliminate women’s engagement in transactional sex altogether [63,70]. This approach may be especially beneficial for women who have experienced ACEs, given their increased risk to transactional sex.

Fourth, the intersection of resilience and gender offers another critical dimension that deserves attention in terms of how we conceptualize and measure resilience. Although this study only recruited young women, and thus was not designed to compare women and men’s experiences, nor did it directly assess the mental health outcomes of ACEs, a meta-analysis of traumatic events in adults found women have a two-fold risk of meeting criteria for PTSD than men [74]. Applying a gender lens to our divergent findings offers new insights as we were able to explore different elements of resilience. Within our photovoice study, participants’ images and descriptions vividly highlighted how gender-specific burdens befell girls in the aftermath of childhood traumas. Steeped in inequitable gender norms, it was the daughters who were expected to leave school, to raise siblings and to subsume other adult responsibilities in the face of childhood neglect, abuse, and orphanhood; burdens that increased their vulnerability to transactional sex. Yet, our quantitative resilience measures were unable to capture these gender dimensions, reflecting a broader critique of the resilience research field that “gender is not well integrated into conceptualizations of resilience.” [75 p461] Developing gender sensitive resilience measures that address the multiple ways that gendered power influences resilience factors, such as gender and social obligations, resource control, and force [30], could add much needed relational and social domain elements to improve how public health researchers operationalize and more broadly understand resilience to promote HIV prevention and women’s health overall.

### Strengths and limitations

Strengths included the mixed methods design and deep theoretical grounding, which enabled a more robust evaluation of resilience within the multi-faceted context of young women’s lives. Through our participatory approach in the qualitative study, we were able to explore the participants’ own definitions and conceptualizations of resilience as it relates to childhood trauma and transactional sexual relationships. Rooting our analysis in the participants’ understanding of resilience guided our interpretation of the findings, which we further validated through regular member checking and triangulation of the photovoice results. In addition, several limitations should be stated. 1) Participants retrospectively self-reported ACEs, which introduced potential recall bias. While this is a valid concern with older adults, collecting ACEs data among adolescents and young adults reduces recall bias due to temporal proximity of the events [76]. 2) Relatedly, participants may have under-reported ACEs as well as transactional relationships—behaviors and experiences that are often associated with social stigma. To decrease the potential for social desirability bias, we used audio-computer assisted self-interview (ACASI) surveys in the quantitative study and diligently emphasized confidentiality throughout the qualitative study to help participants feel more comfortable disclosing sensitive information. 3) The quantitative analysis used a cross-sectional design, and we were thus unable to draw conclusions about causality and temporality between ACEs, transactional sex, and resilience. As the parent study did not comprehensively assess ACEs and resilience at multiple follow-up visits, we were limited in our ability to longitudinally assess how ACEs affect risk of transactional sex over the life course. Future longitudinal cohort studies of this transitional age group should collect ACEs and resilience measures at multiple time points to more fully examine potential moderation and mediation pathways and gain a better understanding of the developmental links between ACE exposure and resilience. 4) Lastly, the data collection of the quantitative and qualitative studies did not occur simultaneously, nor did it include the same participants. Due to COVID-19 imposed movement restrictions, the qualitative data collection was delayed. This resulted in the median age of the HPTN 068 cohort being over 25 years at the time of the photovoice study. Thus, we purposefully sampled a separate group of sexually active young women aged 18–25 years from the same study setting to increase the comparability of the quantitative and qualitative study populations’ life phases.

## Conclusion

Our study utilized a convergent parallel design to ask complementary questions about the interplay between resilience, ACEs, and transactional sex. The high prevalence of both ACEs and transactional sex among young SA women in rural Mpumalanga highlight these issues as ongoing and urgent challenges that need to be addressed. Even as our study was unable to quantitatively show resilience-promoting factors buffered the negative effects of ACEs on transactional sex, the evidence from the photovoice study suggests that resilience was important in women’s lives and influenced their engagement in transactional sex in both positive and negative directions. These dueling tendencies may explain our divergent findings and warrant further investigation. Future research should explore the development of gender sensitive and HIV-specific resilience measures and assess robust social protection program interventions that address the complex gender and power dynamics that relentlessly exacerbate women’s exposure and vulnerability to risky transactional sex and HIV.

## Supporting information

S1 File Inclusivity in global research. (PDF)

S2 File Photovoice ethics and safety handout Xi-Tsonga version available upon request. (PDF)

S3 File Media release form Xi-Tsonga version available upon request. (PDF)

S4 File Photovoice SHOWED group discussion facilitation guide. (PDF)

S1 Table ACE questions. (PDF)
