# Supplementary material for: “My childhood affected my ability to be resilient in both good and bad ways”: A mixed methods examination on the links between adverse childhood experiences, resilience, and transactional sex among young South African women
Source: PLoS One. 2026 Jan 28;21(1):e0341216. doi: 10.1371/journal.pone.0341216 (PMC12851491; doi:10.1371/journal.pone.0341216)
Supplement: S1 File — (PDF) [file pone.0341216.s001.pdf]

Inclusivity in global research

PLOS' policy on inclusivity in global research aims to improve transparency in the reporting of research performed outside of researchers' own country or community and ensures that PLOS publications reporting global research adhere to high standards for research ethics and authorship. Authors of relevant research articles may be asked to complete the questionnaire below, which outlines ethical, cultural, and scientific considerations specific to inclusivity in global research. This questionnaire may be requested when researchers have travelled to a different country to conduct research, if research uses samples collected in another country, research with Indigenous populations or their lands, or if research is on cultural artefacts. Researchers travelling to another country solely to use laboratory equipment will not normally be required to complete the questionnaire. However, the questionnaire can be requested at the journal's discretion for any submission – if you have been requested to complete this questionnaire by the PLOS journal you submitted to, please do so.

Please complete the questionnaire below and include this as a Supporting Information file with your manuscript. Note that if your paper is accepted for publication, this checklist will be published with your article in the supporting information files. Please ensure that you reference the checklist in the main body of your manuscript. We suggest adding a subsection 'Inclusivity in global research' to your Methods section and adding the following sentence: "Additional information regarding the ethical, cultural, and scientific considerations specific to inclusivity in global research is included in the Supporting Information (SX Checklist)"

The questions have been designed to be applicable to a wide range of study types, and there are subsections for both human subjects research and non-human subjects research. If any of the questions are not relevant to your research please mark them as "N/A" as appropriate.

Ethical considerations, permits and authorship

This section is applicable to all research types.
Provide details as to who granted permissions and/or consent for the study to take place in the Methods section of your manuscript. This should include the names of **all** ethics boards, governmental organizations, community leaders or other bodies that provided approval for the study. If individuals provided approval refer to these people by their role or title but do not list their name(s).

Reported on page number: 8-9

"Institutional Review Board approvals for the HPTN 068 Study (UNC IRB #16-0203), Tsima Community Mobilization Study (UNC IRB #14-2214), and all research activities for this mixed methods study, including the merging and analyzing of de-identified data sources and conduct of the photovoice study were obtained from the University of North Carolina at Chapel Hill (UNC IRB #21-0042). Ethics approval was also obtained from the University of Witwatersrand's Human Research Ethics Committee (HREC IRB #320451, #101012), and Mpumalanga Department of Health. The Tsima Community Mobilization Study was additionally approved by the University of California, San Francisco (UCSF IRB #14-13575). The parent randomized control trial, the HPTN 068 Study (trial registration #NCT01233531), adhered to the Declaration of Helsinki's guidelines for biomedical research involving human subjects."

If there were any deviations from the study protocol after approval was obtained please provide details of these changes in the Methods section of your manuscript.

Reported on page number: N/A

Did this study involve local collaborators that are residents of the country where the research was conducted or members of the community studied? If you do not have any authors from said communities, please provide an explanation for this below.

Yes, this study was deeply rooted in community-based and participatory principles, and as such, the study involved local collaboration throughout the planning, execution, and dissemination phases. Three co-authors, including the first author, are South African (SA) citizens or permanent residents, each of whom have lived and worked in SA between 11-35+ years, though none are from the local Bushbuckridge area itself. The photovoice study was carried out by a small three-person study team—comprised of the first author and two field workers who are local community members. This team worked closely with the study site's Public Engagement Office (PEO) staff, which maintains ongoing relationships with elected village Community Development Forums, traditional authorities, and coordinates the monthly Community Advisory Board (CAB) representing all 31 administrative villages.

The study team also worked closely with two local youth partners and a broader time-bound Stakeholder Working Group (SWG) that we established to guide the project through key milestones. The SWG consisted of a sub-set of members from the site's CAB and additional representatives from community-based organizations working in the spaces of HIV prevention, gender-based violence and youth empowerment. These local collaborators provided essential guidance, accountability, and ensured the research remained community-centered. While their contributions were invaluable, these individuals and advisory groups do not meet PLOS authorship criteria and are therefore acknowledged rather than listed as authors.

Finally and critical to this study, the study participants who are local to the community served as key collaborators. Each participant took an active part in shaping the study design and data collection (e.g., jointly developed the photo assignment research question each week) and data analysis (e.g., we convened a coding workshop with the participants which contributed to the thematic analysis; participant-led Community Forum and Dissemination).

Everyone listed as an author should meet PLOS' criteria for authorship and all individuals who meet these criteria should be included in the author byline, rather than the acknowledgements. For further information please see the journal's Authorship Policy.

Human subjects research (e.g. health research, medical research, cross-cultural psychology)

Did you obtain written informed consent from a representative of the local community or region before the research took place? How did you establish who speaks for the community? Details of written informed consent obtained from study participants should be reported separately in the Methods section of your manuscript.

Formal written informed consent processes for the participants are outlined in the Methods section of this manuscript. In addition, we followed South African protocols and procedures for obtaining local community-level consent by submitting and receiving Ethics approvals from the local university-based ethics committee that overseas research in this area and the provincial department of health (see details above). We also engaged with the established Community Advisory Board for this research site and other local leaders (who were referred by the Public Engagement Office staff).

How did members of the local community provide input on the aims of the research investigation, its methodology, and its anticipated outcome(s)?

During formative planning, we convened two Stakeholder Working Group (SWG) consultations to gather community input on study design, implementation considerations, and the feasibility of conducting photovoice in this community. The research team and community members collaboratively developed a recruitment strategy responsive to the sensitive nature of recruiting young women who had experienced childhood trauma (See Methods, p 14). SWG discussions helped identify potential concerns and inform action plans, such as developing a safety protocol for any potential social harms or re-traumatization. Illustrative of our engagement, one of our SWG youth partners who works as an auxiliary social worker reviewed the safety protocol and coordinated a trusted local social worker that she personally vouched for to receive referrals for any potential social harms or other participant needs. Public Engagement Office staff additionally ensured we upheld local cultural norms and ethical practices throughout the study.

When engaging with the local community, how did you ensure that the informed consent documents and other materials could be understood by local stakeholders?

All informed consent documents underwent an iterative development and review process designed to ensure study materials were clear and understandable in both English and the local language of Xitsonga. Consent forms were developed from locally-used templates, reviewed by PEO staff, and assessed for understandability (no jargon). English versions were then translated into Xitsonga by a trained field worker and back-translated by a separate field worker for quality control. Two youth SWG members reviewed both English and Xitsonga versions before finalization and Ethics Committee submission. The SWG also reviewed the recruitment flyer to ensure it was understandable, culturally sensitive, and age-appropriate. Other materials were adopted from existing resources previously developed, vetted, and used in this community (e.g., S1 Fig. Photovoice ethics and safety handout).

Will the findings of the research be made available in an understandable format to stakeholders in the community where the study was conducted (e.g. via a presentation, summary report, copies of publications, etc.)? Please provide details of how this will be achieved.

Yes. We convened a photo exhibit and community forum dissemination event in November 2023 as the final photovoice study activity. Community forums are an established and expected photovoice outcome ensuring adequate dissemination with research communities. The event brought together former participants, SWG members, site leadership, PEO staff, and local HIV implementation partners. Before the event, the study team (first author and two field workers) and participants met privately to review findings and co-develop a facilitation plan while actively reaffirming participants' rights to privacy and anonymity. Given the option, the participants chose to present the findings themselves, sharing displayed photos with illustrative quotes while the study team provided facilitation support. The event generated robust discussion among participants, community stakeholders, and research staff, allowing us to triangulate results and expand findings through stakeholder perspectives (Also reported in Methods, pp 16-17).

Non-human subjects research using specimens/ animals collected as part of the study, or those housed in archival collections. Examples include archaeology, paleontology, botany and zoology.

Did the permission you obtained from a local authority to perform the study include an agreement on access to outputs and benefit sharing? This may include procedures to enable fair distribution of the benefits and resources arising from the research performed. Please include any details of Prior Informed Consent and Benefit Sharing Agreements obtained. These may be required by field-specific regulations, for example the Convention on Biological Diversity (CBD) and the associated Nagoya Protocol.

N/A

If the material used in your study was imported, please A) provide the year it was imported and B) indicate whether permits were obtained to import/export the materials used, C) provide details of any permits obtained. If this information is not available, please indicate this.

N/A

If you used archival specimens, please state how the material used in your study was acquired by the institute it is held in and provide details of any permits obtained for the original excavations/ sample collection. If this information is not available, please indicate this.

N/A

How was the potential cultural significance of the materials collected in your study to local communities considered in your research design? Were Indigenous peoples and/or local researchers and institutions involved with archaeological excavations / collection of specimens? If so, please provide a description of their involvement.

N/A

If your manuscript includes photographs of human remains please indicate whether authors obtained permission from descendants or affiliated cultural communities to do so.

N/A
