# Supplementary material for: “My childhood affected my ability to be resilient in both good and bad ways”: A mixed methods examination on the links between adverse childhood experiences, resilience, and transactional sex among young South African women
Source: PLoS One. 2026 Jan 28;21(1):e0341216. doi: 10.1371/journal.pone.0341216 (PMC12851491; doi:10.1371/journal.pone.0341216)
Supplement: S2 File — (PDF) [file pone.0341216.s002.pdf]

# Photovoice Ethics and Safety

During Photovoice, you will take pictures of activities, events, symbols, and people (photo subjects) that best respond to the framing questions of this research study. This work can have consequences in terms of the safety and wellbeing of you as the photographer, the subject of the photograph and the broader community. Therefore, there are a few "ethics and safety rules" to follow when taking photographs in your community.

### *Protect your personal safety.*

1. "Shooting smart" – maintaining your personal safety – is of highest priority. No photo is worth personal danger.
2. Remember that there are alternative ways to present issues (e.g. through abstract representation).
3. Take your photos in public spaces (from which you can photograph without being seen as trespassing) rather than on private property.
4. Avoid taking photos that could generate conflict or harm for individuals or the community.

### *Protect the subjects of your photographs from harm.*

1. Please respect the privacy of others. If someone does not want their picture taken, don't take it.
2. Please emphasize to photo subjects that the photographs are meant for dissemination (e.g. photos may be used in reports or shown in photograph exhibits in the community).
3. It is essential that photo subjects sign a release form to be photographed. For children or youth under the age of 18, you will need approval from a parent or guardian. This is provided for on the release form. Please ask for more copies if you need them.
4. Photos that show a person's face or any identifying features cannot be shown without a subject's release.

### *Protect the community from potential harm.*

1. Consider if the potential benefits for collective good from the photograph outweigh the potential for both individual and collective harm. For example, does the photograph provide a unique perspective and insights that may increase awareness and understanding of people's daily lives and challenges in your community? Or does it depict stereotypes that could negatively impact the people (photo subjects) or those who live in the community more broadly?

Adapted from: Community Mobilizer's Handbook, Community Mobilization Workshop Manual, and Community Mobilization Toolkit. 2015. Available from: https://genderjustice.org.za/?s=tsima
