# Supplementary material for: “My childhood affected my ability to be resilient in both good and bad ways”: A mixed methods examination on the links between adverse childhood experiences, resilience, and transactional sex among young South African women
Source: PLoS One. 2026 Jan 28;21(1):e0341216. doi: 10.1371/journal.pone.0341216 (PMC12851491; doi:10.1371/journal.pone.0341216)
Supplement: S3 File — (PDF) [file pone.0341216.s003.pdf]

AGINCOURT
Medical Research Council/ Wits University
Rural Public Health and Health Transitions Research Unit

UNC

PHOTO RELEASE FORM

I give and assign to the MRC/Wits-Agincourt Unit, its partners and its nominees the irrevocable right and unlimited permission to use photographs that may include me in presentations, publications, educational materials, photo exhibitions, or for any other similar educational purpose so long as they do not identify me by name or through other background information.

I understand and agree such photographs of me (and Minor) may be shown in public or placed on the Internet. I hereby waive any right to approve that I (and Minor) may have to inspect or approve the final product or products that may be used in connection therewith or the use to which it may be applied.

I HAVE READ, UNDERSTAND AND ACCEPT THE ABOVE PHOTO RELEASE.

Name of person photographed (please print): ____

Age (if under 18): ___________

Street address, village/town, postal code: ____

____

Signature: _________________________________ Date: _______ /_______ / ________

CONSENT OF PARENT OR LEGAL GUARDIAN IF ABOVE INDIVIDUAL IS UNDER AGE 18.

I consent and agree, individually and as parent or legal guardian of the minor name above, to for foregoing terms and provisions. I hereby warrant that I am of full age and have every right to contract for the minor in the above regard. I state further that I have read the above information release and that I am fully familiar with its contents.

Print Name: _______________________________ Relationship: ___________________________________

Signature: _________________________________ Date: _______ /_______ / __________

****

Photographer name: ____

Signature: ___________________________________ Date: _______ /_______ / _________

Assignment / Location: ____
