# Supplementary material for: “My childhood affected my ability to be resilient in both good and bad ways”: A mixed methods examination on the links between adverse childhood experiences, resilience, and transactional sex among young South African women
Source: PLoS One. 2026 Jan 28;21(1):e0341216. doi: 10.1371/journal.pone.0341216 (PMC12851491; doi:10.1371/journal.pone.0341216)
Supplement: S4 File — (PDF) [file pone.0341216.s004.pdf]

Photovoice Follow-up Discussion Guide

Agenda & Facilitator's Guide for
Resilience and HIV Prevention in Young Women in South Africa

**5 minutes**	**Welcome and introduction**<br>❒ Welcome and Introductions<br>❒ Review agenda for session
**5 minutes**	**Review group norms**<br>❒ Review ground rules for the gathering and subsequent photo-discussion groups will help make the gathering a safe, respectful, and comfortable environment for everyone to learn and share.<br>❒ Review each norm and give a brief explanation.
**5 minutes**	**Review SHOWED process**<br>❒ Review how photo-discussion will go<br>❒ Each person will share the photo they took and give an explanation of why you took them and how they relate to the photo assignment.<br>❒ As a group, we will choose 1 "focus" photo from all of the group photos to discuss in-depth<br>❒ We will use the focus photo to go through the SHOWED process, a series of questions that are used to facilitate discussion that moves from a personal level to group analysis to action steps.<br><br>SHOWED questions include:<br>❒ What do we **S**ee in the photo?<br>❒ What is **H**appening in the photo?<br>❒ How does this photo relate to **O**ur lives?<br>❒ **W**hy do these issues exist?<br>❒ How can we become **E**mpowered by our new social understanding? And<br>❒ What can we **D**o to address these issues?
**20 minutes**	**Review research question for this session:**<br>***How has our childhood affected our ability (both good and bad) to be resilient and prevent HIV and pregnancy?***<br><br>**Share photographs taken, and select focus photograph to use for SHOWED.**<br>❒ Review photo assignment for the week.<br>❒ Exercise: "Show & Tell" – Participants show each picture taken for project – explain in a few sentences why they took it.<br>❒ Ask group to: Suggest a picture that they feel best represents the assignment for our photo-discussion – or a photo they would just like to discuss.<br>❒ Choose – ideally using consensus or majority if needed.

**30 minutes**	**SHOWED PROCESS**<br><br>Primary questions and probes to guide the discussion include:<br>1. **What do we See in the photo?**<br>• What objects, things in the picture stand out for you?<br>• What colors do you see?<br>• How does the lighting affect this picture?<br><br>2. **What is Happening in the photo?**<br>• When we see the situation in the image, what is really happening?<br>• If people in the photo, ask: How do you think the person/people felt about [fill in details]?<br>• If relevant, ask: What else is happening in the background?<br><br>3. **How does this photo relate to Our lives?**<br>• How does this image / person's experience remind you of situations you or others have had?<br>• How have you felt when childhood circumstances / trauma affected your resilience (both good and bad ways), and circumstances now as a young adult who is at risk of HIV?<br>• How do you prevent HIV in your life?<br>• <u>*How does resilience help you prevent HIV?*</u><br>• How does resilience help you prevent pregnancy?<br>• Is this a common situation? Could this happen to others? Who?<br><br>4. **Why do these issues exist?**<br>• How do these common childhood traumas (e.g. Losing a parent, abuse, neglect) affect resilience in our community?<br>• How does HIV and pregnancy among young women this affect our community?<br>• Who or what is responsible for perpetuating this situation?<br><br>5. **How can we become Empowered by our new social understanding?**<br>• What insights does this offer us about the issues some young women face?<br>• How are we part of the problem? How can we be part of the solution?<br><br>6. **What can we Do to address these issues?**<br>• What strategies can we come up with to help assure that young women are able to prevent HIV and pregnancy?<br>• How might we advocate for young women who are having a difficult time accessing HIV prevention services or contraception?
**10-20 minutes**	**Summary discussion: Themes, similarities, and differences**<br>LOOKING AGAIN AT ALL THE PHOTOS:<br>❒ Discuss similarities/differences among all the pictures.<br>❒ Record any new, main or prominent themes that emerge from seeing all of the photos together.<br>❒ <u>Brainstorm and agree on photo assignment for next session</u>.<br>❒ Review date and time for next Photo Discussion session.
