# Supplementary material for: “My childhood affected my ability to be resilient in both good and bad ways”: A mixed methods examination on the links between adverse childhood experiences, resilience, and transactional sex among young South African women
Source: PLoS One. 2026 Jan 28;21(1):e0341216. doi: 10.1371/journal.pone.0341216 (PMC12851491; doi:10.1371/journal.pone.0341216)
Supplement: S1 Table — (PDF) [file pone.0341216.s005.pdf]

S1 Table. ACE Questions

ACE	Question
	When you were growing up, during the first 18 years of your life….
Witness emotional abuse	Did you see or hear a parent or household member in your home being yelled at, screamed at, sworn at, insulted or humiliated?
Witness physical abuse	Did you see or hear a parent or household member in your home being slapped, kicked, punched, or beaten up?
	Did you see or hear a parent or household member in your home being hit or cut with an object, such as a stick (or cane), bottle, club, knife, whip etc.?
Experience emotional abuse	Did a parent, guardian, or other household member yell, scream, or swear at you, insult or humiliate you?
	Did a parent, guardian or other household member threaten to, or actually, abandon you or throw you out of the house?
Experience physical abuse	Did a parent, guardian or other household member spank, slap, kick, punch or beat you up?
	Did a parent, guardian or other household member hit or cut you with an object, such as a stick (or cane), bottle, club, knife, whip etc.?
Experience child abuse	Have you ever experienced, witnessed, or been repeatedly confronted with any child abuse (severe beatings, sexual acts with someone 5 years older than you while you were younger than 18, etc.)
Mother death	Is your mother alive? (If no, ask:)<br>How old were you when your mother died?
Father death	Is your father alive? (If no, ask:)<br>How old were you when your father died?

Note on data sources: Questions on abuse are from WHO*'s Adverse Childhood Experiences International Questionnaire.* Questions on parental death collected as part of demographics.
